# α-Lactosylceramide Protects Against iNKT-Mediated Murine Airway Hyperreactivity and Liver Injury Through Competitive Inhibition of Cd1d Binding

**DOI:** 10.3389/fchem.2019.00811

**Published:** 2019-11-28

**Authors:** Alan Chuan-Ying Lai, Po-Yu Chi, Christina Li-Ping Thio, Yun-Chiann Han, Hsien-Neng Kao, Hsiao-Wu Hsieh, Jacquelyn Gervay-Hague, Ya-Jen Chang

**Affiliations:** ^1^Institute of Biomedical Sciences, Academia Sinica, Taipei, Taiwan; ^2^Department of Chemistry, University of California, Davis, Davis, CA, United States

**Keywords:** NKT cell, glycolipid, CD1d, asthma, liver injury, ConA

## Abstract

Invariant natural killer T (iNKT) cells, which are activated by T cell receptor (TCR)-dependent recognition of lipid-based antigens presented by the CD1d molecule, have been shown to participate in the pathogenesis of many diseases, including asthma and liver injury. Previous studies have shown the inhibition of iNKT cell activation using lipid antagonists can attenuate iNKT cell-induced disease pathogenesis. Hence, the development of iNKT cell-targeted glycolipids can facilitate the discovery of new therapeutics. In this study, we synthesized and evaluated α-lactosylceramide (α-LacCer), an α-galactosylceramide (α-GalCer) analog with lactose substitution for the galactose head and a shortened acyl chain in the ceramide tail, toward iNKT cell activation. We demonstrated that α-LacCer was a weak inducer for both mouse and human iNKT cell activation and cytokine production, and the iNKT induction by α-LacCer was CD1d-dependent. However, when co-administered with α-GalCer, α-LacCer inhibited α-GalCer-induced IL-4 and IFN-γ production from iNKT cells. Consequently, α-LacCer also ameliorated both α-GalCer and GSL-1-induced airway hyperreactivity and α-GalCer-induced neutrophilia when co-administered *in vivo*. Furthermore, we were able to inhibit the increases of ConA-induced AST, ALT and IFN-γ serum levels through α-LacCer pre-treatment, suggesting α-LacCer could protect against ConA-induced liver injury. Mechanistically, we discerned that α-LacCer suppressed α-GalCer-stimulated cytokine production through competing for CD1d binding. Since iNKT cells play a critical role in the development of AHR and liver injury, the inhibition of iNKT cell activation by α-LacCer present a possible new approach in treating iNKT cell-mediated diseases.

## Introduction

Invariant natural killer T (iNKT) cells are a rare subset of T cells that possess properties of both conventional T and natural killer (NK) cells (Wu and Van Kaer, 2011; Cameron et al., 2015). Unlike conventional T cells, iNKT cells express a highly restricted T cell receptor (TCR)-α chain (Vα14/Jα18 in mice and Vα24/Jα18 in humans) and a moderately diverse TCR-β chain (Vβ2, Vβ7, and Vβ8 in mice and Vβ11 in humans; Carreno et al., 2016). iNKT cells are activated through invariant TCR by the recognition of lipid-based antigens presented by the MHC class I-like CD1d molecule found on antigen presenting cells (Laurent et al., 2014; Cameron et al., 2015). Activated iNKT cells produce large quantities of cytokines including the Th2 cytokine IL-4 and the Th1 cytokine IFN-γ, which can enhance the function of other immune cells such as NK, B and T cells (Carnaud et al., 1999; Kitamura et al., 2000; Brigl et al., 2003). Therefore, iNKT cells play a key role in bridging the innate and adaptive immunity.

Due to their restricted TCR rearrangements, all iNKT cells share the ability to recognize glycolipids with their sugar head attached in an α-anomeric configuration to the polar head group of a lipid (Kjer-Nielsen et al., 2006). α-galactosylceramide (α-GalCer), also known as KRN7000, is the prototypical glycolipid of this type and has the capability to activate all iNKT cells with a mixed Th1 and Th2 response when presented by CD1d molecule (Kawano et al., 1997; Zhang et al., 2008). α-Glucuronosylceramide (GSL-1), a glycolipid extracted from *Sphingomonas* bacteria, represents another CD1d ligand with α-linked glucuronic acid and a mixture of at least three different sphingosine bases (Perola et al., 2002; Kinjo et al., 2005). Similar to α-GalCer, GSL-1 is also a potent stimulator of iNKT cells, primarily by engaging the TCR expressed by these cells (Perola et al., 2002; Kinjo et al., 2005; Long et al., 2007). Consequently, both α-GalCer and GSL-1 are commonly used to study the function of iNKT cells or iNKT cell-related diseases.

iNKT cells have been implicated in a number of immune-related diseases due to their multi-functional responses. Activated iNKT cells can enhance tumor immunity (Kawano et al., 1999; Aspeslagh et al., 2013) as well as suppress autoimmune disease (Singh et al., 2001). However, they are also associated with the disruption of mucosal homeostasis in the intestines and airways (Nau et al., 2014) and contribute to allergic airway inflammation in various allergen models, including ovalbumin (OVA) and house dust mite (HDM) extract, through Th2-biased cytokine responses (Akbari et al., 2003; Lisbonne et al., 2003; Wingender et al., 2011). Furthermore, both α-GalCer and GSL-1 have been shown to induce airway hyperreactivity (AHR) through iNKT cell activation as glycolipid-induced AHR was abolished in iNKT cell-deficient CD1d^−/−^ (human CD1d analog) and Jα18^−/−^ mice (Meyer et al., 2006). Additionally, iNKT cells also contribute to concavalin A (ConA)-induced hepatitis by inducing liver injury (Kaneko et al., 2000; Takeda et al., 2000). Given their pathogenic roles, the development of lipid antagonist that limit the activation of pathogenic iNKT cells may be of therapeutic relevance.

In this paper, we examined the effects of α-lactosylceramide (α-LacCer), an α-GalCer analog with a lactose head instead of galactose head and a shortened acyl chain in the ceramide tail, on iNKT cells. We found that α-LacCer weakly induced mouse and human iNKT cell activation in a CD1d-dependent manner. However, when co-treated with α-GalCer, α-LacCer suppressed the iNKT cell-activating properties of α-GalCer in terms of IL-4 and IFN-γ production. The therapeutic application of α-LacCer was evaluated *in vivo*, in which this glycolipid effectively attenuated α-GalCer- and GSL-1-induced AHR and Con-A-mediated liver injury. Lastly, our results in the plate-bound CD1d binding assay suggested the inhibitory function of α-LacCer was mediated by competitive CD1d binding.

## Materials and Methods

### Mice

Eight- to ten-week-old C57BL/6 and BALB/c female mice were purchased from National Laboratory Animal Center (Taipei, Taiwan). CD1d^−/−^ (human CD1d analog) mice (Stock No: 002962) were purchased from The Jackson Laboratory (Maine, USA). All animals were housed under specific pathogen-free conditions. This study (13-03-525) was carried out in accordance with the recommendations and guidelines of Academia Sinica Institutional Animal Care and Use Committee (IACUC), and all protocols were approved by the IACUC.

### Reagents

α-GalCer (KRN7000) was purchased from Funakoshi (Tokyo, Japan). RPMI 1640, DMEM, newborn calf serum (NBCS), and red blood cell (RBC) lysis buffer were purchased from Gibco™ (Waltham, MA, USA). Fetal bovine serum (FBS) was purchased from Biological Industries (Beit-Haemek, Israel). Recombinant mouse IL-2 and GM-CSF were purchased from BioLegend (San Diego, CA, USA). Recombinant mouse IL-15, human IL-4, and GM-CSF were purchased from PeproTech (Rocky Hill, NJ, USA). Recombinant human IL-2 was purchased from eBioscience (San Diego, CA, USA).

### *In vitro* Stimulation of Mouse Vα14^+^T Hybridomas, Splenocytes, and iNKT Cells

Vα14^+^T hybridomas (NK1.2), A20 and A20-CD1d cells, which were maintained in cRPMI medium, were kindly provided by Dr. Mitchell Kronenberg (La Jolla Institute, CA). For mouse Vα14^+^T hybridoma stimulation, NK1.2 cells (10^5^) were co-cultured with A20 or A20-CD1d cells (5 × 10^4^) and stimulated with glycolipid. Splenic iNKT cells were expanded with α-GalCer, and CD1d tetramer^+^ TCRβ^+^ cells were sorted by FACSAria (BD Bioscience, San Jose, CA, USA) with a purity of >95%, and sorted iNKT cells were cultured with IL-2 and IL-15 and rested for 24 h before use. For mouse iNKT cell stimulation, sorted mouse iNKT cells (10^5^) were co-cultured with irradiated GM-CSF-treated bone marrow-derived dendritic cells (3 × 10^4^) and before stimulation with glycolipids. Mouse splenocytes (10^6^) were isolated from fresh mouse spleen via mechanical dissociation before stimulation with glycolipids. Neutralization of CD1d was performed with 0.5 μg/well of anti-CD1d antibody (1B1, eBioscience).

### *In vitro* Culture of Human PBMCs and iNKT Cells

Human peripheral blood mononuclear cells (PBMCs) from healthy volunteers were collected from whole blood through gradient-centrifugation with Histopaque-1077 (Sigma-Aldrich). Human iNKT cells were isolated from PBMCs as described previously (Kim et al., 2011). This study (AS-IRB-BM-15054 v.1) was carried out in accordance with the recommendations and guidelines of Academia Sinica Institutional Review Board on Biomedical Science Research (IRB-BM) with written informed consent from all subjects. All subjects gave written informed consent in accordance with the Declaration of Helsinki. The protocol was approved by the IRB-BM. 0.5 μg/well of anti-CD1b antibody (SN13, BioLegend) and anti-CD1d antibody (51.1, BioLegend) were used for blocking CD1b and CD1d, respectively.

### Measurement of Airway Hyperreactivity in Mice

BALB/c mice were intranasally (i.n.) administered with 1 μg of α-GalCer or 10 μg of GSL-1 in the presence or absence of 1 μg α-LacCer for 24 h. Mice were anesthetized with 100 mg/kg in body weight of pentobarbital (Sigma-Aldrich), tracheotomized and mechanically ventilated via the FinePointe RC system (Buxco Research Systems, Wilmington, NC, USA). AHR was assessed by measuring the lung resistance and dynamic compliance in response to increasing doses of aerosolized methacholine (1.25–40 mg/mL, Sigma-Aldrich).

### Collection and Analysis of BALF From Mice

Upon exposure of trachea, the airway was lavaged twice with 1 ml of PBS supplemented with 2% FCS. BALF was pooled, and cells were pelleted by centrifugation and fixed onto slides. The slides were stained with Diff-Quik solution (Polysciences Inc.), and differential cell count was performed.

### Murine Lung Cell Isolation

The whole lungs were flushed by PBS injection (supplemented with 2% FCS) through the right ventricle and minced prior to incubation in 3 ml DMEM medium with 0.1% (v/v) DNase I (Worthington Biochemicals) and 1.6 mg/mL collagenase IV (Worthington Biochemicals) for 40 min at 37°C. Tissues were filtered through a 100 μm mesh to obtain single cell suspensions. Red blood cells were removed by incubating in ACK lysing buffer (GIBCO) for 5 min at 25°C. Single cell suspensions were suspended in the appropriate buffer for further processing.

### Flow Cytometry

Single cell suspensions were stained with fixable viability dye eFluor^®^ 780 (eBioscience) for 30 min at 4°C, and Fc receptors were blocked with anti-CD16/32 (BioLegend) blocking antibody prior to surface staining with antibodies. Antibodies for surface staining used are listed, mouse: CD45 (30-F11, BioLegend), TCR β (H57-597, BioLegend), CD69 (H1.2F3, eBioscience), CD1d tetramer (NIH); human: CD45 (HI30, eBioscience), TCRα/β (IP26, BioLegend), CD69 (FN50, BioLegend), Vα24 (6B11, BD Bioscience). For intracellular staining, single cell suspensions were stimulated with 50 ng/mL phorbol 12-myristate 13-acetate (PMA) (Sigma-Aldrich), 1 μg/mL ionomycin (Sigma-Aldrich) and 1 μg/mL Golgi stop A (BD Biosciences) for 4 h. After surface staining, cells were fixed and permeabilized with Cytofix/Cytoperm solution (BD biosciences) and further stained intracellularly with human IFNγ (B27, BioLegend) and human IL-4 (MP4-25D2, BioLegend). For Ki67 staining, cells were fixed and permeabilized with Cytofix/Cytoperm solution (eBioscience), and further stained with Ki67 (SolA15, eBioscience). Data were acquired using LSR II (BD Biosciences) and analyzed using the FlowJo software (BD Biosciences).

### Murine ConA-Induced Liver Injury Model

BALB/c or CD1^−/−^ mice were injected intravenously (i.v.) with 25 mg/kg ConA (Sigma-Aldrich) for 24 h. In α-LacCer protection experiments, BALB/c mice were pre-treated with 5 μg/ml α-LacCer (i.p.) for 24 h prior to ConA treatment. Serum was collected at 0, 6, and 24 h for aspartate aminotransferase (AST) and alanine aminotransferase (ALT) detection, and livers were harvested at 24 h post-injection for H&E staining.

### Plate-Bound CD1d Binding Assay

96-well strepavidin plates (Thermo scientific) were incubated with 0.5 μg of mouse CD1 or human CD1d monomer protein per well in PBS for 24 h in dark at room temperature. Plate was washed with PBS twice, and indicated glycolipids were loaded and incubated overnight under 37°C. After 24 h, the plate was washed twice, and 10^5^ iNKT cells were added and cultured for 48 h. The supernatant was harvested, and the levels of IFN-γ and IL-4 were detected by ELISA.

### ELISA

Cytokines (mouse IL-2, IL-4, and IFNγ; human IFNγ and IL-4) in culture supernatant, lung lysate, serum, or BALF of mice were analyzed with ELISA kits from Biolegend, with the exception of mouse IL-13 (eBioscience). For the determination of cytokine concentrations *in vivo*, lungs were flushed and minced thoroughly prior to sonication with Bioruptor^®^ Plus sonicator (Diagenode) in RIPA buffer. Lung protein lysate was obtained via centrifugation. To determine cytokine concentrations in the serum, blood samples collected from mice were incubated at room temperature to allow coagulation and the collection of sera.

### Statistics

Data were analyzed with the GraphPad Prism software (GraphPad Prism software, San Diego, CA, USA). Statistical analysis was determined using the Student's two-tailed *t-*test and the two-way analysis of variance (ANOVA).

## Results

### α-LacCer Activates NK1.2 Hybridoma and Mouse iNKT Cells in a CD1d-Dependent Manner

**Figure 1:**
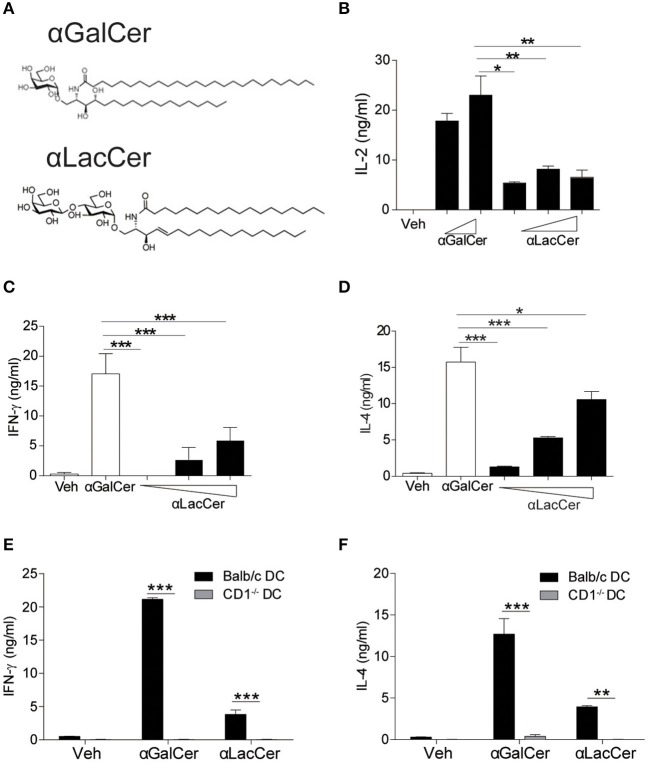
α-LacCer activates NK1.2 hybridoma and mouse iNKT cells in a CD1d-dependent manner. **(A)** The structures of α-galactosyl ceramide (α-GalCer) and its analogs, α-lactosyl ceramide (α-LacCer). **(B)** Vα14^+^ T hybridoma cells (NK1.2) were co-cultured with A20-CD1d cells in the presence of α-GalCer (100, 1,000 ng/ml) or α-LacCer (10, 100, 1,000 ng/ml) for 48 h. The level of IL-2 in the supernatant was measured (*n* = 3–4). **(C,D)** Sorted mouse iNKT cells in co-culture with irradiated BMDCs were stimulated with α-GalCer (100 ng/ml) or α-LacCer (10, 100, 1,000 ng/ml) for 48 h, and the levels of IFN-γ **(C)** and IL-4 **(D)** in the supernatant were measured (*n* = 3). **(E,F)** Sorted mouse iNKT cells in co-culture with irradiated CD1d^−/−^ or WT BMDCs were stimulated with α-GalCer (100 ng/ml) or α-LacCer (100 ng/ml) for 48 h. The levels of IFN-γ **(E)** and IL-4 **(F)** in the supernatant were measured (*n* = 3). Data are representative of three independent experiments and presented as means ± s.e.m. [^*^P < 0.05, ^**^P < 0.01, and ^***^P < 0.001; Student's *t*-test **(B–F)**].

The synthetic protocol of α-LacCer, a new α-GalCer analog with lactose substitution at the galactose head (Hsieh et al., 2014), is shown in Supplementary Figure 1, where a trimethylsilyl protected ceramide was reacted with an activated lactosyl iodide to give the alpha-lactoside in 61% purified yield. Deprotection of the acetate protecting groups quantitatively afforded the desired material for this study. The structures of α-GalCer and α-LacCer are shown in Figure 1A, respectively.

We first assessed their immune-stimulating activities in both Vα14^+^T hybridoma cells (NK1.2) and primary mouse iNKT cells. In comparison to α-GalCer, α-LacCer induced a lower level of IL-2 production by NK1.2 cells (Figure 1B). Similarly, the levels of IFN-γ (Figure 1C) and IL-4 (Figure 1D) produced by α-LacCer-treated iNKT cells co-cultured with DCs were lower than cells treated with α-GalCer. Moreover, mouse iNKT cells failed to produce IFN-γ (Figure 1E) and IL-4 (Figure 1F) in response to both glycolipids when co-cultured with CD1d^−/−^ DCs, suggesting both α-GalCer and α-LacCer induce mouse iNKT cell activation in a CD1d-dependent manner.

### α-LacCer Suppresses α-GalCer-Induced Cytokine Production *in vitro* and *in vivo*

**Figure 2:**
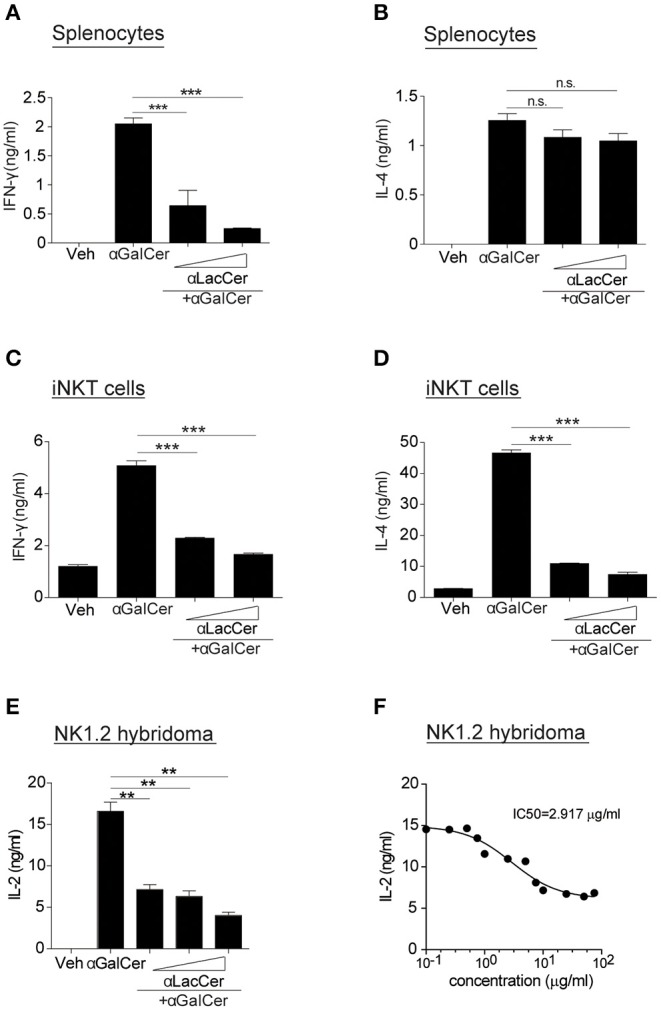
α-LacCer suppresses α-GalCer-induced cytokine production *in vitro*. **(A,B)** Splenocytes from BALB/c mice were stimulated with 100 ng/ml α-GalCer in the presence or absence of α-LacCer (5 or 20 μg/ml) for 48 h. The levels of IFN-γ **(A)** and IL-4 **(B)** in the supernatant were measured (*n* = 3). **(C,D)** Sorted mouse iNKT cells were co-cultured with irradiated BMDCs and stimulated with α-GalCer (100 ng/ml) in the presence or absence of α-LacCer (5 or 20 μg/ml) for 48 h. The levels of IFN-γ **(C)** and IL-4 **(D)** in the supernatant were measured (*n* = 3). **(E)** NK1.2 cells in co-culture with A20-CD1d cells were stimulated with 100 ng/ml α-GalCer in the presence or absence of α-LacCer (100, 1,000, or 10,000 ng/ml) for 48 h. The level of IL-2 in supernatants was measured (*n* = 3). **(F)** NK1.2 cells in co-culture with A20-CD1d cells were stimulated with 100 ng/ml α-GalCer in the presence of incremental levels of α-LacCer (0.1–100 μg/ml) for 48 h. The level of IL-2 in supernatants was measured (*n* = 4), and GraphPad Prism was utilized for calculating the IC50. Data are representative of three independent experiments and presented as means ± s.e.m. [n.s., not significant; ^**^P < 0.01 and ^***^P < 0.001; Student's *t*-test **(A–F)**].

Since α-LacCer has been shown as a CD1d ligand weakly inducing iNKT cell activation, we theorized α-LacCer might be a good candidate to suppress iNKT cell activation by competing for CD1d-binding. To prove this hypothesis, we co-treated splenocytes with α-GalCer and α-LacCer. We observed that α-LacCer co-treatment reduced α-GalCer-induced IFN-γ (Figure 2A) and IL-4 (Figure 2B) production by mouse splenocytes. Additionally, α-LacCer inhibited α-GalCer-induced IL-2 production by NK1.2 hybridomas (Figure 2C), as well as IFN-γ (Figure 2D) and IL-4 (Figure 2E) production by mouse iNKT cells co-cultured with DCs. To further determine the inhibitory characteristic of α-LacCer, we performed a functional assay using NK1.2 hybridomas and measured the IC50 as 2.917 μg/ml against α-GalCer (Figure 2F).

**Figure 3:**
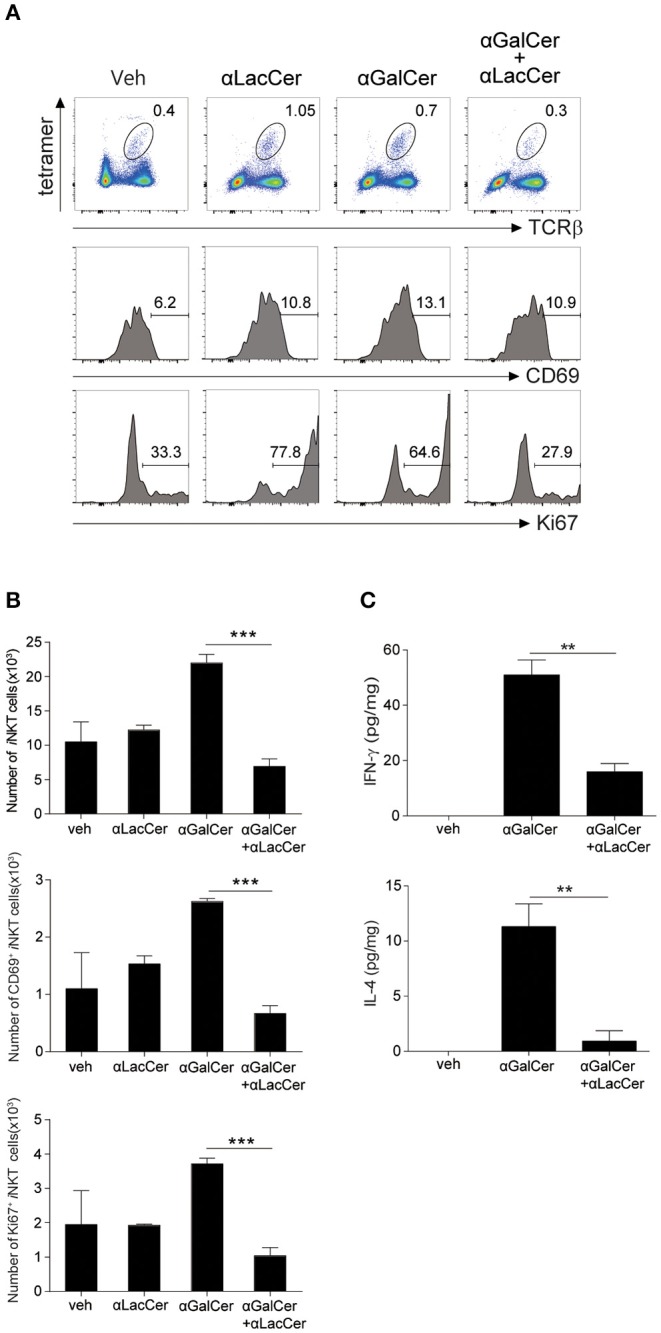
α-GalCer-induced iNKT cell activation and proliferation are suppressed by α-LacCer *in vivo*. BALB/c mice were injected intraperitoneally with 1 μg of α-GalCer and α-LacCer, and the lungs were harvested 3 days post-injection. **(A)** Flow cytometry analysis of CD69 and Ki67 expressions in iNKT cells (CD45^+^TCRβ^+^CD1d tetramer^+^ cells). **(B)** Absolute numbers of total, CD69^+^, and Ki67^+^ iNKT cells calculated from the flow cytometry data (*n* = 3). **(C)** IFN-γ and IL-4 levels in the lung lysates were measured by ELISA (*n* = 3). Data are representative of three independent experiments and presented as means ± s.e.m. [^**^P < 0.01 and ^***^P < 0.001, Student's *t*-test **(B,C)**].

To determine the inhibitory effect of α-LacCer *in vivo*, BALB/c mice were injected intraperitoneally with 1 μg α-GalCer in the presence or absence of 1 μg α-LacCer. We found that α-LacCer co-treatment reduced α-GalCer-induced lung iNKT cell activation (Figure 3A) and proliferation (Figure 3B), as determined by the expressions of CD69 and Ki67, respectively. Furthermore, mice receiving α-GalCer and α-LacCer showed lower levels of lung IFN-γ and IL-4 comparing to α-GalCer-treated mice (Figure 3C). Taken together, these data indicate that α-LacCer suppress α-GalCer-induced iNKT activation, proliferation and cytokine production *in vitro* and *in vivo*.

### α-LacCer Inhibits α-GalCer-Induced Lung iNKT Cell Activation and Airway Hyperreactivity (AHR)

**Figure 4:**
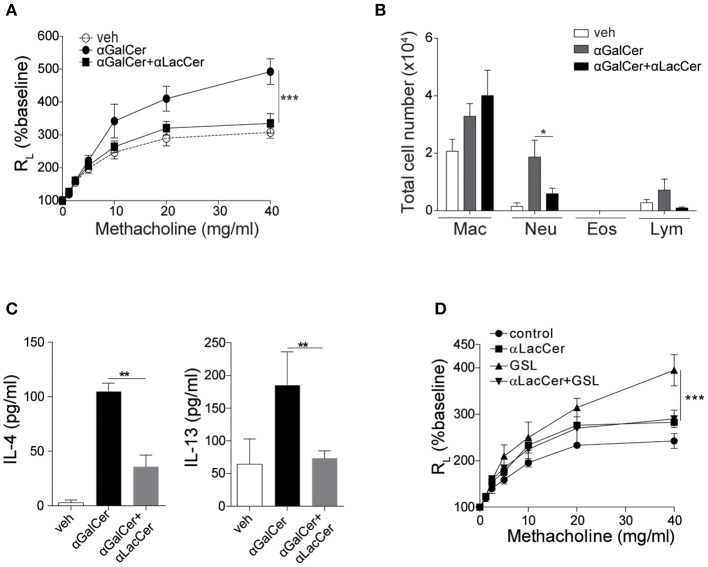
α-LacCer suppresses α-GalCer and GSL-1-induced airway hyperreactivity. BALB/c mice were treated intranasally with 1 μg α-GalCer **(A–C)** or 10 μg GSL-1 **(D)** in the presence or absence of 1 μg α-LacCer for 24 h. **(A,D)** Changes in lung resistance (R_L_) in response to increasing doses of methacholine [*n* = 6–8/group in **(A)**; *n* = 3–5/group in **(D)**]. **(B)** The numbers of macrophage (Mac), neutrophil (Neu), eosinophil (Eos), and lymphocyte (Lym) in the BALF (*n* = 7–8). **(C)** The levels of IL-4 and IL-13 in the lungs assessed using ELISA (*n* = 4). Data are representative of three independent experiments and presented as means ± s.e.m. [^*^P < 0.05, ^**^P < 0.01, and ^***^P < 0.001; Two-way ANOVA **(A,D)** and Student's *t*-test **(B,C)**].

Previous studies have shown that activation of lung iNKT cells by α-GalCer mediated the development of AHR and airway inflammation (Meyer et al., 2006; Wingender et al., 2011). Thus, we wondered whether α-LacCer could suppress α-GalCer-induced AHR. We treated mice intranasally with α-GalCer in the presence or absence of α-LacCer for 24 h. We found α-LacCer co-treatment attenuated α-GalCer-induced AHR (Figure 4A) and neutrophil infiltration in the bronchoalveolar lavage fluid (BALF) (Figure 4B). Accordingly, α-GalCer-induced IL-4 and IL-13 production were significantly inhibited by α-LacCer (Figure 4C). Similar suppression was observed in the GSL-1-induced AHR model (Figure 4D), further highlighting the therapeutic potential of α-LacCer for the treatment of asthma.

### α-LacCer Protects Against ConA-Induced Liver Injury

**Figure 5:**
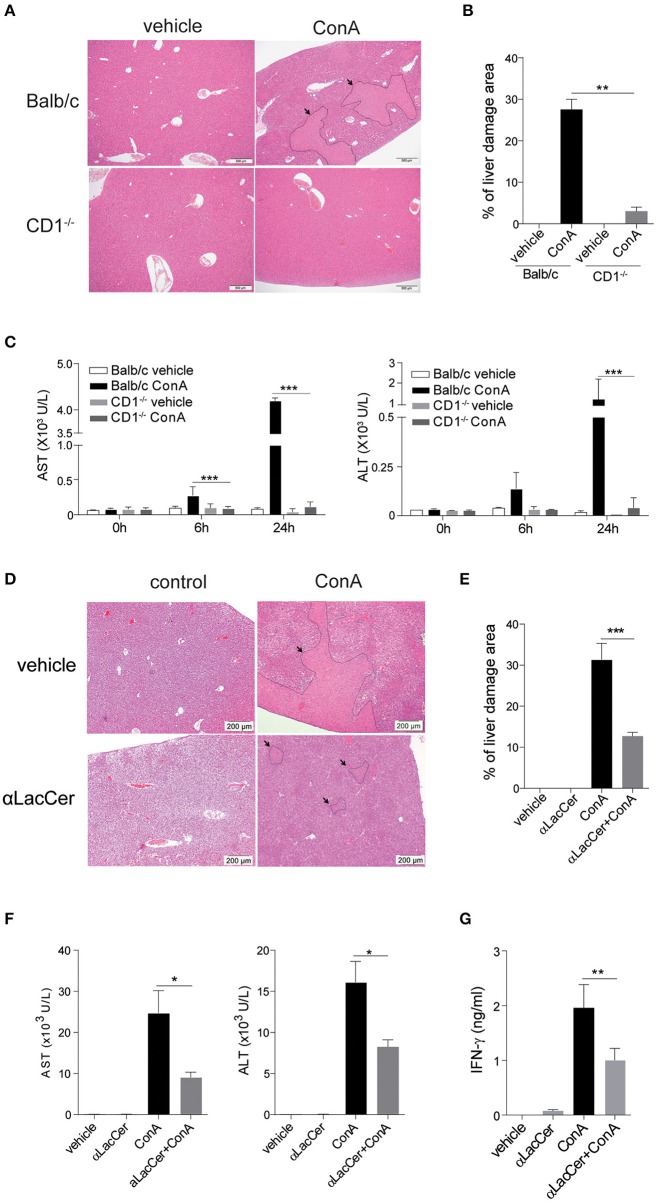
α-LacCer protects against ConA-induced liver injury. **(A–C)** ConA was injected intravenously (25 mg/kg) into BALB/c (*n* = 3) or CD1d^−/−^ mice (*n* = 3). Sera were collected at 0, 6, and 24 h post-injection, and mice were sacrificed at 24 h post-injection. **(A)** H&E stained liver sections (Scale bar = 500 μm). **(B)** Percentage of liver damage area. **(C)** Serum levels of ALT and AST. **(D–G)** BALB/c mice were first pre-treated intraperitoneally with α-LacCer (5 μg/ml) for 24 h and followed by ConA (25 mg/kg; i.v.) administration. Sera and livers were harvested 24 h post-ConA treatment. **(D)** H&E stained liver sections (Scale bar = 200 μm). **(E)** Percentage of liver damage area (*n* = 3). **(F)** Serum levels of AST and ALT (*n* = 4). **(G)** The serum IFN-γ level measured by ELISA (*n* = 4). Data are representative of three independent experiments and are presented as means ± s.e.m. [^*^P < 0.05, ^**^P < 0.01, and ^***^P < 0.001; Student's *t*-test **(B,C,E–G)**].

iNKT cells have been shown to participate in ConA-induced liver damage (Mattner, 2013). Hence, we examined whether α-LacCer could protect against iNKT cell-mediated liver damage. In accordance to a previous study (Takeda et al., 2000), ConA induced liver damage in WT, but not iNKT cell-deficient CD1d^−/−^ mice (Figures 5A,B). Likewise, serum levels of alanine aminotransferase (ALT) and aspartate aminotransferase (AST) were markedly lower in ConA-treated CD1d^−/−^ mice compared to their WT counterpart (Figure 5C), implying that ConA-induced hepatitis was iNKT cell-dependent.

We next examined whether the pre-treatment with α-LacCer could protect against ConA-induced liver damage. α-LacCer was injected intraperitoneally 24 h before ConA injection, and the extent of liver damage was measured. We found that α-LacCer pre-treatment significantly reduced the percentage of liver damage area (Figures 5D,E). Similarly, ConA-induced AST, ALT (Figure 5F), and IFN-γ (Figure 5G) production in sera were suppressed by α-LacCer. These data demonstrated the protective effect of α-LacCer against ConA-induced liver injury.

### α-LacCer Suppresses α-GalCer-Induced Activation and Cytokine Production by Human iNKT Cells

**Figure 6:**
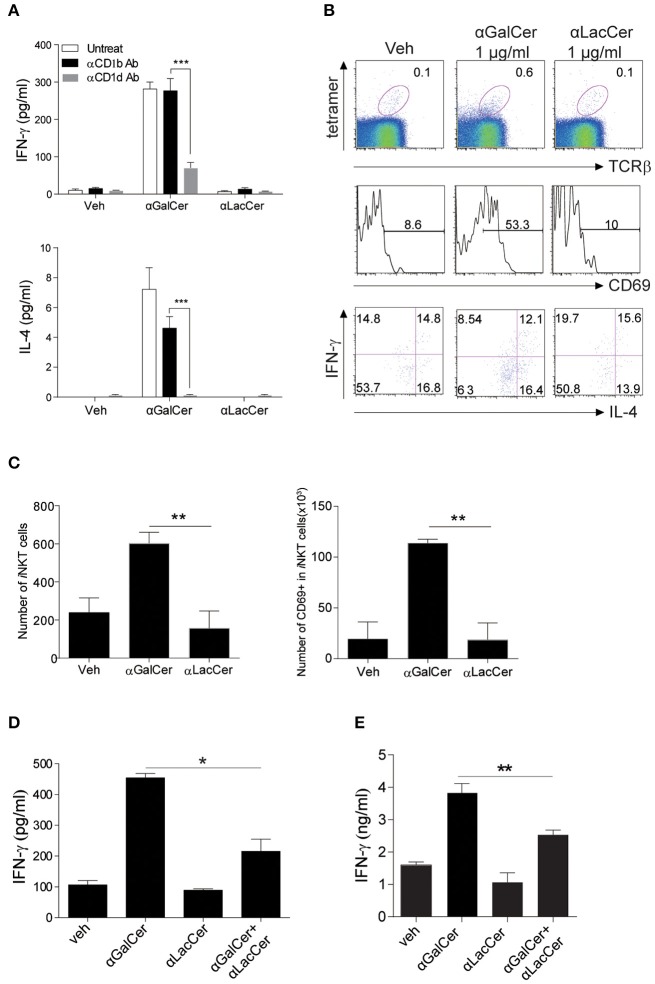
α-LacCer suppresses α-GalCer-induced cytokine production by human iNKT cells *in vitro*. **(A)** Sorted human iNKT cells were stimulated with α-GalCer and α-LacCer (1 μg/ml) in the absence or presence of anti-CD1b or anti-CD1d antibody for 72 h. The levels of IFN-γ and IL-4 in the supernatant were analyzed (*n* = 3–6). **(B,C)** Human PBMCs were cultured with 1 μg/ml α-GalCer and α-LacCer for 7 days. **(B)** FACS analysis of CD69, IFN-γ, and IL-4 expressions in human iNKT cells (CD45^+^tetra-CD1d^+^TCRβ^+^). **(C)** Absolute numbers of total and CD69^+^ iNKT cells. **(D)** Human PBMCs were stimulated with α-GalCer (100 ng/ml) in the presence or absence of α-LacCer (1 μg/ml) for 3 days. The level of IFN-γ was analyzed (*n* = 3). **(E)** Human iNKT cells were cultured with irradiated DCs pre-treated with 100 ng/ml α-GalCer in the absence or presence of 1 μg/ml α-LacCer for 3 days. The levels of IFN-γ and IL-4 were analyzed (*n* = 3–5). Data are representative of three independent experiments and presented as means ± s.e.m. [*n.s*., not significant; ^*^P < 0.05, ^**^P < 0.01, and ^***^P < 0.001; Student's *t*-test **(A,C–E)**].

To examine the possible effects of α-LacCer in human, human iNKT cells were isolated and co-cultured with irradiated DCs with α-GalCer or α-LacCer in the presence or absence of anti-human CD1b or anti-human CD1d antibody. Human iNKT cells showed little to no induction of IFN-γ and IL-4 when treated with α-LacCer comparing to α-GalCer (Figure 6A). Additionally, the induction was CD1d-dependent as blocking CD1d, but not CD1b, impaired cytokine production induced by α-GalCer. α-LacCer also failed to induce iNKT cell proliferation (Ki67^+^ cells) and activation (CD69^+^ cells) in terms of percentage (Figure 6B) and frequency (Figure 6C). Furthermore, α-LacCer co-treatment significantly suppressed α-GalCer-induced IFN-γ secretion from human PBMC (Figure 6D) and human iNKT cells (Figure 6E). Overall, these data indicated that α-LacCer effectively inhibited both human and mouse iNKT cell activation and cytokine production.

### α-LacCer Suppresses α-GalCer-Stimulated Cytokine Production by iNKT Cells by Competing for CD1d Binding

**Figure 7:**
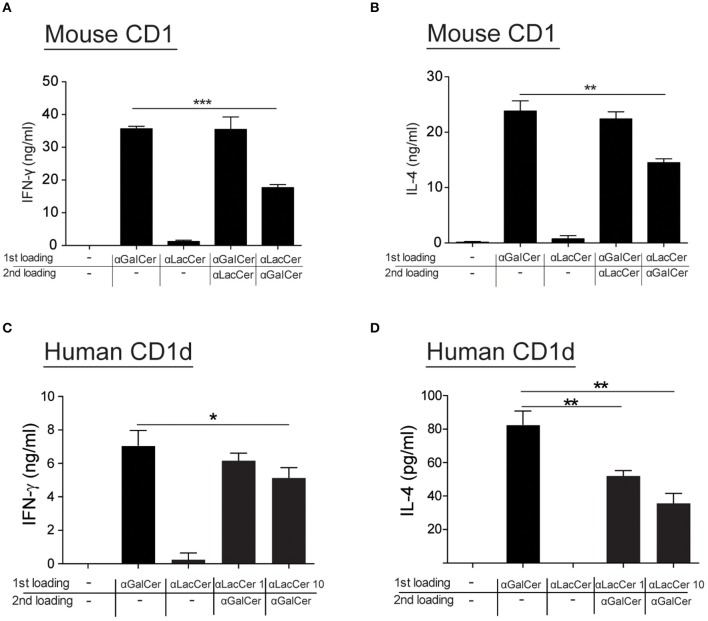
α-LacCer suppresses α-GalCer-stimulated cytokine production by competing for CD1d binding. **(A,B)** Sorted mouse iNKT cells were cultured in the CD1-coated plate pre-loaded with 100 ng/ml α-GalCer or 1 μg/ml α-LacCer with or without the second loading of α-GalCer or α-LacCer for 48 h. The levels of IFN-γ **(A)** and IL-4 **(B)** in the culture supernatant were assessed (*n* = 3). **(C,D)** Sorted human iNKT cells were cultured in the CD1d-coated plate pre-loaded with α-LacCer (1 or 10 μg/ml) with the second loading of α-GalCer (100 ng/ml) for 48 h. The levels of IFN-γ **(C)** and IL-4 **(D)** in the supernatant were assessed (*n* = 3–6). Data are representative of three independent experiments and are presented as means ± s.e.m. [^*^P < 0.05, ^**^P < 0.01, and ^***^P < 0.001; Student's *t*-test **(A–D)**].

Since α-LacCer could be docked into both mouse CD1-iNKT TCR and human CD1d-iNKT TCR complexes (Data not shown), we hypothesized that the suppressive effect exhibited by α-LacCer toward α-GalCer might be due to the competitive binding to CD1d. We examined this hypothesis by performing the cell-free plate-bound CD1d assay. We pre-loaded mouse CD1d and human CD1d-coated plates with α-GalCer or α-LacCer, followed by a second loading with α-LacCer or α-GalCer, respectively. Pre-loading with α-LacCer reduced α-GalCer-induced IFN-γ (Figure 7A) and IL-4 (Figure 7B) production in mouse iNKT cells. Similar results were obtained with human iNKT cells (Figures 7C,D). In short, our results suggested that α-LacCer suppressed α-GalCer-stimulated cytokine production by mouse and human iNKT cells by competing for CD1d binding.

## Disucssion

In this study, we demonstrated that α-LacCer is a weak activator of mouse and human iNKT cells comparing to α-GalCer. Yet, when co-administered with α-GalCer *in vitro*, α-LacCer suppressed α-GalCer-induced mouse and human iNKT cell activation and cytokine production. Through a NK1.2 hybridoma-based functional assay, we measured the IC50 of α-LacCer as 2.917 μg/ml against α-GalCer. When co-administered with α-GalCer or GSL-1 *in vivo*, α-LacCer suppressed AHR development by both glycolipids, as well as α-GalCer-induced neutrophilic inflammation by inhibiting lung iNKT cell activation and cytokine production. Furthermore, α-LacCer protected against ConA-induced liver injury in a CD1d-dependent manner. Lastly, pre-loaded α-LacCer inhibited α-GalCer-stimulated IL-4 and IFN-γ production by iNKT cells in the plate-bound CD1d binding assay, suggesting the inhibitory effect of α-LacCer is due to its competition for CD1d binding. Results from this study highlighted the potential therapeutic utility of α-LacCer for the treatment of iNKT cell-associated diseases.

iNKT cells are associated with various allergic and non-allergic diseases. For instance, iNKT cells contribute to the induction of pathology in mucosal tissues of the airways and the gastrointestinal tract and drive allergic inflammatory reactions through the production of Th2 cytokines, as well as the recruitment and degranulation of mast cells and eosinophils (Nau et al., 2014). iNKT cells also contribute to lupus development by augmenting Th1-biased immune responses and autoantibody secretion (Zeng et al., 2003). Additionally, collagen-induced arthritis (CIA) is associated with iNKT cells because CIA can be ameliorated by blocking the interaction between CD1d and iNKT cells using anti-CD1d neutralizing antibody (Chiba et al., 2005). Furthermore, α-GalCer treatment in mice often leads to detrimental side effects, resulting in disease exacerbation rather than protection (Wu and Van Kaer, 2009). Hence, the pathogenic roles of activated iNKT cells in various diseases justify the need to develop new and improved therapeutic approaches targeting these cells.

Several lipids have been shown to suppress iNKT cell activation, but none have been developed pharmacologically. For instance, An et al. revealed that GSL-Bf717, a bacterial glycol sphingolipids extracted from *Bacteroides fragilis*, did not activate iNKT but could inhibit iNKT hybridoma activation when treated together with α-GalCer (An et al., 2014). In another study, 1,2-dipalmitoyl-sn-glycero-3-phosphoethanolamine-N[methoxy(polyethyleneglycol)-350 (DPPE-PEG_350_) was developed and reported to be effective in inhibiting iNKT cell activation and attenuating the development of allergen-induced AHR (Lombardi et al., 2010) and atherosclerosis (Li et al., 2016). Furthermore, a non-lipid based antagonist, *Griffonia simplicifolia*-derived isolectin B4 (IB4), has been shown to inhibit the sphingolipid isoglobotrihexosylceramide (iGb3)-induced iNKT cell stimulation by binding to the terminal Gal α1,3 Gal of iGb3, thus preventing its recognition by mouse Vα14 and human Vα24 iNKT cells (Keusch et al., 2000; Zhou et al., 2004). Hence, our findings add to the growing list of iNKT cell antagonists that can be exploited for therapeutic purposes. Mechanistically, the inhibitory function of α-LacCer on α-GalCer activity seems to involve the competitive binding of CD1d. However, this does not mean α-LacCer prevents the activation of iNKT through the inhibition of CD1d function. Rather, α-LacCer could still induce iNKT activation through CD1d while it is occupying the binding site, but at a much weaker level comparing to α-GalCer. This speculation is supported by the *in vitro* data showing that α-LacCer could activate iNKT in a CD1d-dependent manner, but with a much lower intensity in comparison to α-GalCer. However, the exact mechanism may require further investigation to reveal.

A similar glycolipid with lactose head, also called α-LacCer, was previously synthesized and found to activate both human and mouse iNKT cells as effectively as α-GalCer in terms of inducing IL-4, but not IFN-γ production (Zhang et al., 2008). This Th2-biased glycolipid exhibited both antitumor and anti-autoimmune disease effects, with greater potency than α-GalCer in ameliorating the latter disease. Contrary to this, the α-LacCer synthesized for our study is a weak iNKT cell activator when compared with the prototypical α-GalCer, as evidenced by the lower IFN-γ and IL-4 production, as well as the lack of iNKT cell expansion and activation when administered *in vivo*. The difference in potency is likely due to the different acyl chain length. The reported α-LacCer retains the 26-carbon long acyl chain found in the prototypical α-GalCer, whereas the α-LacCer synthesized in our study is 8 carbons shorter (C18). Truncation of the acyl chain has previously been shown to reduce the binding stability of glycolipids to CD1d without affecting TCR affinity (McCarthy et al., 2007). While this may explain the weaker activating property of our α-LacCer, it does not justify the observation that α-LacCer can effectively suppress α-GalCer-induced iNKT cell activation and the downstream effects through competitive CD1d binding. Hence, the difference in the activation potency between the reported α-LacCer and ours may be caused by other factors, and additional studies will be required to unravel the mechanisms involved.

We observed that α-LacCer effectively suppressed ConA-induced liver injury. Although it has been suggested that ConA-induced liver injury is CD1d-independent (Zeissig et al., 2017), a recent study reported that CD1d-mediated activation of iNKT cells is required for further activation of these cells in response to ConA (Wei et al., 2016). In their study, the authors demonstrated the importance of gut bacteria-derived glycolipids in driving hepatic iNKT cell activation. When comparing to specific pathogen-free mice, germ-free mice had lower levels of gut bacteria-derived glycolipids and were resistant to ConA-induced liver injury. Based on this finding, we postulate that α-LacCer may reduce ConA-induced liver damage by preventing the initial iNKT cell activation.

In summary, the data indicate that even though α-LacCer failed to potently activate iNKT cells, it did effectively suppress the iNKT cell-activating properties of α-GalCer both *in vitro* and *in vivo*. Moreover, α-LacCer attenuated α-GalCer, and GSL-1-induced AHR and ConA-triggered liver injury. As determined by the plate-bound CD1d binding assay, the pre-loading of α-LacCer prior to α-GalCer treatment attenuated the stimulatory properties of α-GalCer, suggesting that α-LacCer competed with α-GalCer for CD1d binding. In conclusion, this study characterized the biological function of α-LacCer highlighting the use of this glycolipid for the clinical treatment of iNKT cell-associated diseases.

## Data Availability Statement

The datasets generated for this study are available on request to the corresponding author.

## Ethics Statement

The animal study was reviewed and approved by Institutional Animal Care and Use Committee of Academia Sinica (AS IACUC), which is affiliated to Academia Sinica.

## Author Contributions

AL planned and performed experiments and wrote the manuscript. P-YC performed the AHR experiments and assisted with experiments. CT assisted with experiments and helped writing the manuscript. Y-CH and H-NK performed the *in vitro* experiments. H-WH and JG-H synthesized and supplied glycolipids. Y-JC conceived and initiated the project, planned experiments, and wrote the manuscript.

### Conflict of Interest

The authors declare that the research was conducted in the absence of any commercial or financial relationships that could be construed as a potential conflict of interest.
